# Mining GO Annotations for Improving Annotation Consistency

**DOI:** 10.1371/journal.pone.0040519

**Published:** 2012-07-25

**Authors:** Daniel Faria, Andreas Schlicker, Catia Pesquita, Hugo Bastos, António E. N. Ferreira, Mario Albrecht, André O. Falcão

**Affiliations:** 1 Department of Informatics, Faculty of Sciences, University of Lisbon, Lisbon, Portugal; 2 Division of Molecular Carcinogenesis, The Netherlands Cancer Institute, Amsterdam, The Netherlands; 3 Department of Computational Biology and Applied Algorithmics, Max Planck Institute for Informatics, Saarbrücken, Germany; 4 Department of Informatics, Faculty of Sciences, University of Lisbon, Lisbon, Portugal; 5 Department of Informatics, Faculty of Sciences, University of Lisbon, Lisbon, Portugal; 6 Centre of Chemistry and Biochemistry, Department of Chemistry and Biochemistry, Faculty of Sciences, University of Lisbon, Lisbon, Portugal; 7 Department of Computational Biology and Applied Algorithmics, Max Planck Institute for Informatics, Saarbrücken, Germany; 8 Department of Bioinformatics, Institute of Biometrics and Medical Informatics, University Medicine Greifswald, Greifswald, Germany; 9 Department of Informatics, Faculty of Sciences, University of Lisbon, Lisbon, Portugal; University College Dublin, Ireland

## Abstract

Despite the structure and objectivity provided by the Gene Ontology (GO), the annotation of proteins is a complex task that is subject to errors and inconsistencies. Electronically inferred annotations in particular are widely considered unreliable. However, given that manual curation of all GO annotations is unfeasible, it is imperative to improve the quality of electronically inferred annotations. In this work, we analyze the full GO molecular function annotation of UniProtKB proteins, and discuss some of the issues that affect their quality, focusing particularly on the lack of annotation consistency. Based on our analysis, we estimate that 64% of the UniProtKB proteins are incompletely annotated, and that inconsistent annotations affect 83% of the protein functions and at least 23% of the proteins. Additionally, we present and evaluate a data mining algorithm, based on the association rule learning methodology, for identifying implicit relationships between molecular function terms. The goal of this algorithm is to assist GO curators in updating GO and correcting and preventing inconsistent annotations. Our algorithm predicted 501 relationships with an estimated precision of 94%, whereas the basic association rule learning methodology predicted 12,352 relationships with a precision below 9%.

## Introduction

The foundation of the Gene Ontology (GO) Consortium was a critical step toward the adoption of formal and objective knowledge representations in biological sciences and addressed the need for knowledge sharing and functional comparisons in the face of the rapid growth of genomic sequence data [1].

GO is currently the *de facto* standard for functional annotation of gene products in the categories molecular function, biological process, and cellular component. The ontology is under constant development because both our knowledge of biological phenomena and our ability to represent that knowledge are continuously growing [2]. While the ontology development is carried out by human curators, it can be assisted by computational approaches that facilitate handling the increasing size and complexity. In this context, the use of the association rule learning methodology has been proposed to identify relationships between GO terms with the goal of enriching the ontology [3], [4]. More recently, Alterovitz et al. proposed a novel method that uses information theory to automatically organize the structure of GO and optimize the distribution of its information [5]. Additionally, the ongoing extension of GO with computable logical definitions will enable the partial automation of the development of the ontology and facilitate the identification of errors and missing relationships [6].

The annotation of UniProtKB [7] proteins with GO terms is carried out by the Gene Ontology Annotation resource (GOA) [8]. Currently, over 98% of the annotations in GOA are inferred electronically, typically from entries in external databases such as InterPro [9]. These annotations are generally considered unreliable as most are based on sequence alignments, which are prone to make and propagate annotation errors [10], [11]. As a result, they are discarded in many studies involving GO annotations. However, it is clear that experimental determination of the function of all proteins or manual curation of all annotations are beyond present resources [8], [10], [12]. Given that electronically inferred annotations play an essential role in providing annotation coverage for an increasing fraction of proteins, it is imperative to assess and improve their quality.

We can consider three types of problems that affect annotation quality: erroneous annotations, incomplete annotations and inconsistent annotations. A protein is erroneously annotated if it was predicted to perform a function that it does not actually perform, and thus is annotated to terms that do not correspond to its true function [10], [11]. A protein is incompletely annotated if its annotations do not describe all its functional aspects in full detail, typically because those aspects have not been fully determined [13]. Two or more proteins are inconsistently annotated if they perform the exact same function but are not annotated to the same terms, likely due to different annotation criteria [14], [15]. In the case of electronically inferred annotations, the fact that proteins can receive annotations from multiple data sources may also lead to inconsistent annotations if those sources are not well coordinated.

In this work, we analyze the molecular function annotations of UniProtKB proteins and estimate the fraction of incomplete and inconsistent annotations, discussing possible strategies to prevent the latter. Finally, we present a data mining algorithm, based on association rule learning, for identifying relationships between molecular function terms. This algorithm can both improve annotation consistency and assist GO curators in developing the ontology.

## Materials and Methods

### The Gene Ontology

The Gene Ontology (GO) is an ontology for describing the function of gene products at the cellular level in three categories: molecular function, biological process, and cellular component. Each of these categories is organized as a directed acyclic graph, with ‘is a’ and ‘part of’ relationships forming its basic structure.

A GO annotation is the association of a gene or protein with a GO term describing one aspect of its function. Each protein may be annotated to multiple terms within each GO category. Each annotation is labeled with an evidence code which indicates the type of evidence upon which the annotation is based (e.g. electronically inferred annotations have the evidence code IEA). Annotations obey the true path rule, meaning that if a protein is annotated with a term, it is also annotated to all its ancestors in the ontology by inheritance.

In this work, we focused only on the molecular function category of GO.

### Dataset

This work is based on release 4.2 of the FunSimMat database, a comprehensive database of semantic and functional similarity values [16]. It compiles protein and annotation data from the UniProtKB [7], annotation data from GOA [8], and ontology data from GO [1], as of the respective releases of October 2010. The database comprises 7.3 million annotated UniProtKB proteins, 21 million annotations, of which 99.4% are inferred electronically, and 8,889 non-obsolete molecular function terms.

To account for small differences between the annotations in GOA and UniProtKB, FunSimMat combines both sets of annotations. However, there are only 24,000 annotations present in UniProtKB that are not present in GOA, so this combination does not have a significant statistical impact in our analysis.

### Molecular Function Classes

A molecular function class (or MFclass) is a unique set of one or more molecular function terms that is used to annotate one or more proteins [16]. Each MFclass corresponds to a representation of a protein function on the molecular function annotation space. For instance, the function of hemoglobin can be described by the MFclass {*oxygen transporter activity*; *heme binding*; *oxygen binding*}.

The concept of MFclass is extremely useful to reduce the dimension of the annotation space because there are many proteins with identical annotations, which are redundant from an annotation perspective. Indeed, the 7.3 million annotated proteins correspond to only 45,244 distinct MFclasses (not counting differences due to redundant annotations).

### Quantifying Redundant Annotations

An annotation is redundant if it is implied by another more specific annotation of the same protein, by the true path rule. For instance, the term *iron ion binding* is a parent of *heme binding* in the molecular function graph. Thus, if a protein is annotated to both *heme binding* and *iron ion binding*, the latter annotation is redundant as it is already implied by the former annotation.

We quantified the fraction of redundant molecular function annotations by computing all annotations that were implied by other annotations. For each annotation {*P_i_*,*t_i_*} where *P_i_* is a protein and *t_i_* a term, we checked whether there exists an annotation {*P_i_*,*t_j_*} such that *t_j_* is a descendent of *t_i_* in the molecular function graph (and therefore implies *t_i_*).

### Quantifying Incomplete Annotations

A protein is incompletely annotated if its annotations do not fully describe its function.

Technically, almost any protein can be incompletely annotated, as few proteins have been studied extensively enough to rule out the possibility that they perform additional functions. In practice, however, we can only distinguish between proteins that have annotations to specific terms which describe detailed functional aspects (e.g. *cytochrome-c oxidase activity*) and proteins that have annotations to generic terms (e.g. *catalytic activity*). Thus, our practical definition is that a protein is incompletely annotated if it has at least one (non-redundant) annotation to a generic term.

In order to quantify incomplete annotations we must first define what is a generic term. It is clear that leaf terms should not be considered generic, since they correspond to the highest level of specificity within the limits of the ontology. However, there are cases of non-leaf terms which describe detailed functional aspects and should also not be considered generic. For instance, the term *iron ion binding* is the most specific term describing a direct interaction with an iron ion, despite having the child term *heme binding*. To account for these cases, we considered that only terms with more than 10 descendents were generic, which correspond to the 5% most generic terms of the molecular function ontology.

### Quantifying Inconsistent Annotations

Inconsistent annotations are cases where proteins that perform the same function are not annotated to the same set of molecular function terms, and thus belong to different, but typically similar, MFclasses. For instance, not all hemoglobins are annotated to *oxygen binding*, which leads to two similar MFclasses: {*oxygen transporter activity*; *heme binding*; *oxygen binding*} and {*oxygen transporter activity*; *heme binding*}. It is evident that the two MFclasses describe the same function since *oxygen binding* is implicitly implied by *oxygen transporter activity*. This is a common form of inconsistent annotations, with a primary (or function-defining) term which implicitly implies a secondary term that is present in some proteins and absent in others (e.g. *ATPase activity* ⇒ *ATP binding*). However, not all cases of inconsistent annotations are as straightforward to detect. For instance, some hemoglobins lack the term *oxygen transporter activity*, and can only be identified as hemoglobins by the presence of the biological process term *oxygen transport*.

The fact that cases of similar functions abound in nature means that identifying inconsistent annotations automatically is extremely difficult. Without manual analysis, it is all but impossible to determine if a pair of similar MFclasses correspond to similar but distinct functions or inconsistent annotations of a single function. For instance, hemerythrins are oxygen-transporting proteins which lack the heme group but use iron as a cofactor, and so their MFclass {*oxygen transporter activity*; *iron ion binding*; *oxygen binding*} is very similar to the main hemoglobin MFclass (note that *heme binding* is a direct child of *iron ion binding*). Thus, any similarity-based criteria that would correctly identify the actual inconsistent hemoglobins would also erroneously identify the hemerythrins as inconsistent hemoglobins.

Unfortunately, the approach proposed by Dolan *et al* to estimate annotation consistency by comparing the annotations of orthologous proteins [15] is not applicable to our dataset. Beyond the daunting task of finding orthologs for all annotated UniProtKB proteins is the problem of data circularity, since most electronic annotations are based on sequence similarity in the first place.

Having tested several similarity-based algorithms for identifying inconsistent annotations without obtaining a reasonable precision, we opted for a manual analysis to estimate the fraction of inconsistent annotations. We selected the 100 most popular MFclasses that correspond to complete annotations and contain at least two molecular function terms, which represent approximately 1 million annotated proteins. For each of these MFclasses, we looked for cases of evident inconsistent annotations by identifying the primary term (or terms) and searching the database for other MFclasses that included that term but differed in secondary terms. We searched for information about each term and protein function in the GO definitions, in ENZYME [17] and InterPro.

### Association Rule Learning

Association rule learning (ARL) is a data mining methodology for uncovering relations between variables in large databases based on their co-occurrence [18]. Given a list of variables and a database of occurrences, an association rule is defined as an implication of the form A
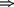
B, where A and B are disjoint sets of one or more variables. The support measures the prevalence of each association and is defined as the fraction of occurrences in the database that include both A and B. The confidence measures the strength of each association and is defined as the fraction of the occurrences including A that also include B (which is an estimation of the conditional probability of B given A). ARL extracts from the database all association rules that are above selected support and confidence thresholds.

In this work, we employ ARL to find relationships between molecular function terms. In particular, we are interested in capturing implicit relationships between aspects of a single function (e.g. *ATPase activity* 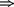 *ATP binding*). This is a simple ARL problem in terms of solution space, since we are only interested in rules between individual terms.

The main goal of the support threshold is to filter out fortuitous associations and ensure statistical significance. Since annotations are deliberate events, we do not need a high threshold for this application of ARL. Therefore, we chose a support threshold of 10 co-annotations. The confidence threshold is more important since it measures the strength of the relationship and we are interested in capturing universal relationships. While this equates to a high confidence threshold, the possibility of inconsistent annotations means that we cannot expect related terms to always occur together. Thus, we selected a confidence threshold of 80%.

### GO Relationship Learning

Applying ARL to GO leads to two problems as the basic methodology is unprepared to deal with structured data [19].

The first problem is the upward propagation of redundant relationships due to the true path rule. If a given relationship *t_1_* 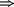 *t_2_* is retrieved by ARL, then all relationships of the form *t_1_* 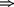 *t_A2_*, where *t_A2_* is an ancestor of *t_2_*, will also be retrieved. This happens because each ancestor *t_A2_* inherits all annotations of *t_2_* and so will have at least as many co-annotations with *t_1_* as *t_2_* does. Consequently, all relationships *t_1_* 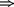 *t_A2_* will have a support and confidence equal to or greater than those of the relationship *t_1_* 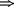 *t_2_*. Additionally, if *t_A1_* is an ancestor of *t_1_* which has few other descendents and/or these have few annotations in comparison with *t_1_*, then the relationship *t_A1_* 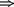 *t_2_* will likely also be retrieved by ARL. To circumvent this problem, we filtered out all redundant relationships, selecting only the most specific. For each relationship *t_1_* 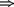 *t_2_*, we excluded all relationships of the forms *t_1_* 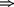 *t_A2_*, *t_A1_* 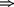 *t_2_* and *t_A1_* 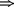 *t_A2_*.

The second problem is the prediction of spurious relationships due to the fact that two GO terms can have shared descendents. If two terms *t_1_* and *t_2_* have a shared child *t_3_* which is frequently annotated, then ARL will likely retrieve a relationship between *t_1_* and *t_2_* in one direction or both. This happens because all annotations of *t_3_* correspond to co-annotations of *t_1_* and *t_2_*. Thus, any relationship between *t_1_* and *t_2_* is likely spurious, since the true relationships are already represented in the ontology (*t_3_* 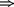 *t_1_* and *t_3_* 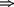 *t_2_*). To address this problem, we excluded relationships between terms that have a descendent distance of 4 or less edges, defining descendent distance as the minimum number of edges that connects two terms passing by one of their shared descendents.

We also made three additions to the ARL methodology to improve the performance of our GO relationship learning (GRL) algorithm, taking into account the nature of the data and the type of relationship we are interested in capturing.

The first of these additions was the exclusion of relationships that include generic terms (i.e. terms with more than 10 descendents). These relationships are common since a high fraction of the electronically inferred annotations are generic, but they are not very useful in the context of our work.

The second addition was the exclusion of relationships between terms that have an ancestral distance of 4 or less edges, defining ancestral distance as the minimum number of edges that connects two terms passing by one of their common ancestors. We verified that relationships between such closely related terms typically correspond to cases of bifunctional proteins (e.g. *asparagine-tRNA ligase activity* 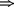 *aspartate-tRNA ligase activity*) or less often to cases of partially synonymous terms (e.g. *proton-transporting ATPase activity, rotational mechanism* 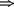 *hydrogen ion transporting ATP synthase activity, rotational mechanism*). Neither case corresponds to an implicit relationship between aspects of a single function, which is the type of relationship we are interested in capturing.

The third addition was the introduction of a novel parameter called agreement, which is formally identical to the confidence, but based on the number of MFclasses that support the relationship rather than the number of individual proteins. We noted that many of the false relationships predicted by ARL corresponded to cases where a common combination of terms overshadows a less common yet biologically meaningful exception. For instance, the relationship *oxygen transporter activity* 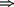 *heme binding* has a high confidence (83%) due to the prevalence of hemoglobin, but there are oxygen transporters which do not have a heme cofactor, such as hemerythrin and hemocyanin. The addition of the agreement increases the sensitivity of ARL to such cases, since we are requiring relationships to not only be true for most known proteins but also for most known functions. The underlying assumption is that when there are few distinct exceptions to a relationship, these are more likely to correspond to inconsistent annotations, whereas when there are many distinct exceptions these are more likely to be biologically meaningful. We selected an agreement threshold of 80% in conjunction with the confidence threshold of 80%.

We computed the support, confidence, agreement, descendent distance, and ancestral distance parameters directly in SQL, from the annotation table, the MFclass table and the GO ‘graph_path’ table. We selected the relationships that met the thresholds for all parameters, then identified and excluded those that were redundant.

We evaluated manually the top 100 GRL relationships ranked by support. For each relationship, we searched for evidences supporting or refuting it in GO, ENZYME, InterPro, Brenda [20], and when necessary in the bibliography. Relationships were classified as true if supporting evidence was found; as reverse if the relationship was true in the reverse direction; as unknown if evidence was inconclusive; and as false if at least one counterexample is known or the relationship is logically impossible.

## Results and Discussion

### Redundant and Incomplete Annotations

The fraction of redundant molecular function annotations (i.e. annotations implied by other annotations according to the true-path rule) was 38% in the FunSimMat release used for our study. While individually redundant annotations are a minor issue, this high fraction has significant impact on the size of the database. There are cases where the redundant annotation has a stronger evidence code than the more specific annotation, and as such cannot be discarded. However, there are few reasons to keep redundant annotations that are inferred electronically, and discarding these would reduce the fraction of redundant annotations to less than 1%.

Based on the criterion that a protein is incompletely annotated if it has any non-redundant annotation to a term with more than 10 descendents, we estimate that 64% of the proteins are incompletely annotated and 68% of the MFclasses correspond to incomplete protein functions. This high fraction is not unexpected, as it reflects the fact that most electronically inferred annotations are generic. However, it should be noted that electronically inferred annotations account for over 1.5 million proteins annotated in detail (i.e. exclusively to leaf terms) with most of the detailed annotations coming from relatively reliable data sources such as InterPro. It is also worth mentioning that 37% of the molecular function leaf terms are currently not annotated to a single protein, which suggests that there is a considerable lag between updates to GO and annotation updates. Thus, some annotations may be incomplete because they are outdated and were based on a mapping to a less detailed version of the ontology.

### Inconsistent annotations

Our manual MFclass analysis revealed that, for 83% of the MFclasses, there was at least one similar MFclass describing the same function. Thus, we estimate that 83% of the protein functions have inconsistent annotations, and at least 23% of the annotated proteins are inconsistently annotated, assuming that the most popular MFclass is the correct representation of each function. The results of the analysis are available in Table S1.

An example of the inconsistencies found is the cytochrome-c oxidase complex, which is represented by at least the 4 MFclasses: {*cytochrome-c oxidase activity*}, {*cytochrome-c oxidase activity*; *electron carrier activity*}, {*cytochrome-c oxidase activity*; *electron carrier activity*; *heme binding*} and {*cytochrome-c oxidase activity*; *electron carrier activity*; *heme binding*; *copper ion binding*}. Note that the term *cytochrome-c oxidase activity* ‘is a’ *heme-copper terminal oxidase activity*, and therefore logically implies *heme binding* and *copper ion binding*. Since the cytochrome-c oxidase complex is involved in electron transport, the term *electron carrier activity* is also implied. Thus, in this case there are three secondary terms implicitly implied by the primary term *cytochrome-c oxidase activity*.

Our analysis focused precisely in finding inconsistent annotations that followed this pattern, with a primary term that implicitly implies one or more secondary terms which are present in some proteins and absent in others. However, we also found cases of major inconsistency which led to ambiguous functions, such as the MFclass {*two-component sensor activity*; *two-component response regulator activity*; *ATP binding*}. The terms *two-component sensor activity* and *two-component response regulator activity* are supposedly incompatible, since a two-component system is formed by a protein that acts as a sensor and another protein that acts as a response regulator (as detailed in the definitions for the corresponding GO terms). Thus, it is unclear whether the MFclass corresponds to misannotated sensors, response regulators, or both.

Cases like this are extreme, but even the more common minor inconsistencies that were the focus of our analysis undermine the very purpose for which GO was created. If proteins aren't annotated consistently, then we can't compare their functions accurately or find proteins with functions of interest reliably. For instance, comparing the MFclasses {*cytochrome-c oxidase activity*} and {*cytochrome-c oxidase activity*; *electron carrier activity*; *heme binding*; *copper ion binding*} results in a low similarity value, even though they correspond to the same function. Likewise, searching for proteins involved in electron transport would fail to retrieve the 15,000 cytochrome-c oxidases which lack the term *electron carrier activity*.

### Implicit *binding* terms

Our MFclass analysis revealed that in most cases of implicit relationships, the secondary terms were *binding* terms describing enzyme-substrate or enzyme-cofactor interactions implied by a primary *catalytic activity* term. There was also an analogous case where the secondary term was a *binding* term describing a transporter-target interaction implied by a primary *transporter activity* term. The fact that *binding* terms are involved in so many implicit relationships is not surprising, since most molecular functions involve interactions with some type of molecule. However, the fact that so many inconsistent annotations arise from these relationships merits further analysis.

The GO annotation conventions state that enzyme-substrate and transporter-target interactions are an implicit part of the catalytic or transporter activity, and therefore co-annotations with the corresponding binding terms are redundant. Thus, we would expect co-annotations of this type to be almost non-existent. The problem is that information about substrates and targets is available in GO only in human-readable definitions of the *catalytic activity* and *transporter activity* terms. As such, *binding* co-annotations are necessary to represent this information in a form amenable to computation. For instance, co-annotating ATPases with *ATP binding* is the only way to explicitly record that these are proteins that interact with ATP so that, for example, a query searching for ATP-binding proteins would retrieve them. Inconsistent annotations arise from the fact that some annotation sources (namely InterPro) opt for this type of co-annotation whereas others follow the annotation conventions.

One solution to this problem would be to represent these interactions explicitly in the ontology as relationships between the *catalytic activity* or *transporter activity* term and the *binding* term corresponding to the substrate or target. This would make the co-annotations with the *binding* terms actually redundant, and would prevent inconsistent annotations, since the *binding* term would always be present by the true-path rule. However, this solution would change the scope of the molecular function ontology by dividing functions into steps or parts and would require the addition of a large number of *binding* terms.

An alternative solution may be provided by the ongoing extension of GO with computable logical definitions [6]. If, as planned, the definitions of *catalytic activity* terms include a list of substrates, then co-annotation with the corresponding *binding* terms would be unnecessary to capture this information in a form amenable to computation. However, this does not make the *binding* co-annotations actually redundant, so annotation sources would have to adapt to the GO update and drop these co-annotations. Thus, it may become necessary to enforce stricter annotation guidelines to prevent inconsistent annotations.

Unlike enzyme-substrate and transporter-target interactions, enzyme-cofactor interactions are not considered redundant by the GO annotation conventions, likely because information about cofactors is not available in GO in any form. However, the distinction between substrates and cofactors is somewhat artificial, considering that molecules such as NAD and FAD can be considered cofactors in some reactions and substrates in others Furthermore, information about cofactors is typically available in the corresponding ENZYME entry for the EC family upon which the GO term was based. Thus, for enzymatic reactions that have a universal cofactor, there is no apparent reason why information about the cofactors should not be available in GO. Nevertheless, the fact that not all cofactors are universal means that inconsistent annotations of this type should be corrected with care.

### Finding implicit relationships between molecular function terms

Given that many cases of inconsistent annotations are tied to implicit relationships between terms, finding such relationships is an important step toward correcting and/or preventing inconsistent annotations. For instance, if we identify the relationship *ATPase activity* 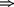 *ATP binding* as universal, then any two MFclasses that include the former term and differ only in the presence of the latter term can automatically be considered as representing the same function. To correct the inconsistency, the co-annotation with *ATP binding* can be removed from all proteins annotated with *ATPase activity* because this co-annotation is redundant under the current GO annotation conventions. Finally, the relationship can be set as a negative annotation guideline in order to prevent future inconsistent annotations.

We applied the association rule learning (ARL) methodology to find these implicit relationships between molecular function terms, with the goal of assisting GO curators in improving annotation consistency and in updating the ontology. ARL has been previously applied to GO with the goal of enriching the ontology [3], [4] or guiding the annotation process [21]. However, these applications were based on relatively small annotation datasets and thus were not significantly affected by the limitations of the ARL methodology. Given that the same was not true for our dataset, we introduced several additions to the ARL methodology in our GO relationship learning (GRL) algorithm, both to address these limitations and to improve its performance (as detailed in the methods section).

Out of the 242,921 pairwise combinations of co-occurring molecular function terms, the basic ARL methodology retrieves 12,352 relationships with a support of 10 co-annotations and a confidence of 80%. However, the large majority of these relationships are either false or uninteresting for our goal, as 77% include generic terms and an additional 5% are relationships between closely related terms (with either close shared descendents or close shared ancestors). Furthermore, half of the remaining relationships are redundant, which is a hindrance since all retrieved relationships must be manually reviewed. Thus, considering that all these relationships are not relevant, the precision of the basic ARL methodology is at most 9% (the top 100 ARL relationships ranked by support are shown in Table S2, to illustrate their low usefulness).

Our GRL algorithm excludes all of these unwanted relationships, which improves its precision and facilitates the task of the GO curators it is meant to assist. Additionally, the introduction of the agreement parameter enables the algorithm to select relationships that are true for most functions in addition to being true for most proteins. Thus, it can exclude false relationships where a prevalent combination of terms overshadows a biologically meaningful exception. Using an agreement threshold of 80% in addition to the confidence threshold of 80% and support threshold of 10 co-annotations, the GRL algorithm retrieved 550 relationships.

We estimated the precision of the GRL algorithm by manually analyzing the top 100 relationships ranked by support (the first 20 evaluation results are shown in Table 1, and the full evaluation is available in Table S3). We found that 92 of these relationships were true, 2 were true but in the reverse direction, 4 were unknown, and 2 were false. Given that our methodology is intended to assist GO curators, for whom it should be easy to identify the correct direction of the relationship, we can consider the reverse direction as true. Thus, if we assume that unknown relationships are false, we obtain an estimated precision of 94%, which indicates that our algorithm is sufficiently precise to be of assistance to GO curators.

**Table 1:** Manual evaluation of the 20 most supported rules selected by our GO relationship learning algorithm.

Subject Term	Predicate Term	Support	Confidence	Agreement	Evaluation
GTPase activity	GTP binding	62218	100%	95%	True
ribonucleoside binding	DNA-directed RNA polymerase activity	18893	100%	100%	Reverse
DNA topoisomerase (ATP-hydrolyzing) activity	ATP binding	18778	97%	82%	True
phosphopantetheine binding	acyl carrier activity	8433	100%	94%	Reverse
1-aminocyclopropane-1-carboxylate synthase activity	pyridoxal phosphate binding	7101	100%	97%	True
adenylate kinase activity	ATP binding	5514	99%	86%	True
tRNA dihydrouridine synthase activity	FAD binding	4559	100%	100%	True
5-formyltetrahydrofolate cyclo-ligase activity	ATP binding	4427	100%	94%	True
glycine-tRNA ligase activity	ATP binding	4073	99%	88%	True
holo-[acyl-carrier-protein] synthase activity	magnesium ion binding	4017	100%	89%	True
arginine-tRNA ligase activity	ATP binding	4005	99%	88%	True
cysteine synthase activity	pyridoxal phosphate binding	4001	100%	97%	True
copper-exporting ATPase activity	ATP binding	3993	99%	89%	True
shikimate kinase activity	ATP binding	3947	99%	91%	True
histidine-tRNA ligase activity	ATP binding	3692	99%	83%	True
alanine-tRNA ligase activity	ATP binding	3630	99%	82%	True
tetrahydrofolylpolyglutamate synthase activity	ATP binding	3585	100%	91%	True
cysteine desulfurase activity	pyridoxal phosphate binding	3512	98%	80%	True
D-alanine-D-alanine ligase activity	ATP binding	3477	99%	85%	True
lysine-tRNA ligase activity	ATP binding	3460	99%	82%	True

Each association is classified as: true if evidence for a relationship between the terms was found; reverse if the reverse rule is true; unknown if no conclusive evidence was found for or against the association; and false if a counterexample was found. The support is given in number of co-annotations.

Since we do not know *a priori* how many implicit relationships exist between molecular function terms, we cannot estimate the recall of our algorithm. However, it should be noted that the introduction of the agreement parameter leads to the exclusion of some relationships of interest. This suggests that the agreement threshold could be lowered to increase the number of relationships captured while maintaining a reasonable precision.

It is notable that 51 of the true (and reverse) relationships analyzed involved a catalytic activity term and a binding term corresponding to a substrate of that activity, such as ATP or NAD. In most of these cases, the GO definition for the catalytic term consists of the chemical equation for the catalyzed reaction, which includes the substrate indicated by the binding term. This reinforces the fact that this information is available in GO, but not in a machine-readable form.

In addition to the enzyme-substrate interactions, there were 36 associations where binding terms were used to describe enzyme-cofactor interactions, with cofactors such as pyridoxal phosphate, FAD or metal ions. Unlike substrates, cofactors cannot currently be found in the GO definitions for catalytic activity, so our algorithm could be useful to identify universal cofactors to be included in logical definitions, particularly in cases where those cofactors are not listed in ENZYME, which was the case in 14 of the relationships analyzed. One curious observation we made regarding cofactors is that although magnesium and manganese ions can be used interchangeably as cofactors by many enzymes, this is not usually reflected in their annotations. Most enzymes are only annotated with the binding term for one of the metals but not both, despite both being listed as possible cofactors in ENZYME or Brenda. Again, these are cases that would be corrected if cofactor information was readily available in GO.

It is interesting to note that one of the false relationships retrieved by our methodology is due to extensive inconsistent annotations. This relationship is actually true for the proteins that are annotated with the subject term, but those annotations are inconsistent. On a first analysis it may seem true that *molybdenum ion transmembrane transporter activity* is associated with *ATP binding* as all known prokaryotic molybdenum transporters are ABC transporters. However, we can verify in InterPro that prokaryotic molybdenum ABC transporters have two subunits: an ABC subunit, which binds (and hydrolyses) ATP, and a permease subunit, which does not. Therefore, we would expect that only 50% of the proteins annotated with *molybdenum ion transmembrane transporter activity* would be co-annotated with *ATP binding*. Instead, we observe this for 100% of the proteins because only the ABC subunits are annotated with *molybdenum ion transmembrane transporter activity*. The permease subunits are annotated with *molybdate ion transmembrane transporter activity* instead, which is probably more accurate considering that molybdate is the only soluble molybdenum ion. In any case, it seems evident that both subunits should be annotated with the same transporter activity term to improve annotation consistency. Additionally, it should be verified whether it is necessary to maintain both transporter activity terms in GO.

## Conclusions

Our analysis of the GOA molecular function annotations revealed that 38% of the annotations are redundant and that the large majority of these are electronically inferred. While redundant annotations are not a major issue, the cost of maintaining such a fraction of redundant information is not irrelevant. Since there are few reasons for maintaining redundant electronically inferred annotations, they could likely be discarded.

Our analysis also revealed that, as expected, the majority of the electronically inferred annotations are relatively generic. However, this may be partially due to a lag between GO updates and annotation sources, since 37% of the molecular function leaf terms are currently not in use for annotating proteins. In spite of this, electronically inferred annotations still account for 1.5 million proteins annotated in detail from relatively reliable data sources.

One issue that deeply affects the quality of electronically inferred annotations is the lack of consistency, as we estimated that there are inconsistencies for 83% of the protein functions and that at least 23% of the proteins are inconsistently annotated. While our analysis focused only on minor inconsistencies tied to implicit relationships between terms, we also detected more severe cases where the function of the proteins could not be clearly identified from their annotations. Furthermore, even minor inconsistencies hinder the usefulness of GO, since they make functional comparisons inaccurate and functional queries unreliable.

We found that many inconsistent annotations corresponded to cases where a *binding* term was used to describe a substrate or cofactor implied by a *catalytic activity* term or a target implied by a *transporter activity* term. The co-annotation with *binding* terms to describe enzyme-substrate or transporter-target interactions is discouraged by the GO annotation guidelines, since it is considered an implicit aspect of the catalytic or transporter activity. However, information about substrates and targets is not available in GO in a computer-accessible form, so representing it with *binding* co-annotations is a way to enable computation. Thus, inconsistent annotations arise from the fact that some annotation sources follow the GO annotation guidelines whereas others do not. The ongoing extension of GO aims to address this issue by representing information about substrates and targets explicitly in the form of computable logical definitions [6]. Nevertheless, the use of *binding* terms to capture this information will continue to be an easy solution for annotation sources unless it is further discouraged. Additionally, it is clear that information about cofactors, which is currently not available in GO in any form, should also be available in the form of computable logical definitions, at least for the catalytic activities which have a universal cofactor.

Given that many cases of inconsistent annotations are tied to implicit relationships between molecular function terms, finding these relationships could be useful both to assist GO curators in updating the ontology and in correcting and preventing inconsistent annotations. Our GO relationship learning (GRL) algorithm was able to find 550 relationships with an estimated precision of 94%. In comparison, the basic association rule learning methodology found 12,352 relationships but with a precision below 9%. Our manual evaluation of the retrieved relationships reinforces the need to represent knowledge about substrates and cofactors explicitly in GO as 51% of the relationships analyzed corresponded to enzyme-substrate interactions and 36% to enzyme-cofactor interactions. While some of these interactions are evident, our algorithm can help identify those that are less evident such as enzyme-cofactor interactions that are not currently listed in ENZYME.

## Supporting Information

Table S1 Inconsistencies related to the 100 most popular multiple-term MFclasses, found upon manual analysis.
(XLS)

Table S2 Top 100 results ranked by support of the basic ARL methodology with a confidence threshold of 80% and a support threshold of 10 proteins.
(XLS)

Table S3 Manual evaluation of the top 100 results of the GRL algorithm, ranked by support. Each association is classified as: true if evidence for a relationship between the terms was found; reverse if the reverse rule is true; unknown if no conclusive evidence was found for or against the association; and false if a counterexample was found.
(XLS)

## References

[1] The Gene Ontology Consortium (2000). Gene ontology: tool for the unification of biology.. Nature Genet.

[2] Leonelli S, Diehl AD, Christie KR, Harris MA, Lomax J (2011). How the Gene Ontology Evolves.. BMC Bioinformatics.

[3] Kumar A, Smith B, Borgelt C (2004). Dependence relationships between Gene Ontology terms based on TIGR gene product annotations.. 3rd International Workshop on Computational Terminology.

[4] Bodenreider O, Aubry M, Burgun A (2005). Non-lexical approaches to identifying associative relationships in the Gene Ontology.. Pac Symp Biocomput.

[5] Alterovitz G, Xiang M, Hill DP, Lomax J, Liu J (2010). Ontology engineering.. Nat Biotechnol.

[6] Mungall CJ, Bada M, Berardini TZ, Deegan J, Ireland A (2010). Cross-Product Extensions of the Gene Ontology.. J Biomed Inf.

[7] The UniProt Consortium (2010). The Universal Protein Resource (UniProt) in 2010.. Nucleic Acids Res.

[8] Barrell D, Dimmer E, Huntley RP, Binns D, O'Donovan C (2009). The GOA database in 2009–an integrated Gene Ontology Annotation resource.. Nucleic Acids Res.

[9] Hunter S, Apweiler R, Attwood TK, Bairoch A, Bateman A (2009). InterPro: the integrative protein signature database.. Nucleic Acids Res.

[10] Devos D, Valencia A (2001). Intrinsic errors in genome annotation.. Trends Genet.

[11] Jones CE, Brown AL, Baumann U (2007). Estimating the annotation error rate of curated GO database sequence annotations.. BMC Bioinformatics.

[12] Friedberg I (2006). Automated protein function prediction - the genomic challenge.. Brief Bioinform.

[13] Huttenhower C, Hibbs MA, Myers CL, Caudy AA, Hess DC (2009). The impact of incomplete knowledge on evaluation: an experimental benchmark for protein function prediction.. Bioinformatics.

[14] Camon EB, Barrell DG, Dimmer EC, Lee V, Magrane M (2005). An evaluation of GO annotation retrieval for BioCreAtIvE and GOA.. BMC Bioinformatics.

[15] Dolan ME, Ni L, Camon E, Blake JA (2005). A procedure for assessing GO annotation consistency.. Bioinformatics.

[16] Schlicker A, Albrecht M (2010). FunSimMat update: new features for exploring functional similarity.. Nucleic Acids Res.

[17] Bairoch A (2000). The ENZYME database in 2000.. Nucleic Acids Res.

[18] Agrawal R, Imielinski T, Swami A (1993). Mining Association Rules Between Sets of Items in Large Databases.. SIGMOD Conference.

[19] Srikant R, Agrawal R (1995). Mining generalized association rules..

[20] Scheer M, Grote A, Chang A, Schomburg I, Munaretto C (2011). BRENDA, the enzyme information system in 2011.. Nucleic Acids Res.

[21] Bada M, Turi D, McEntire R, Stevens R (2004). Using reasoning to guide annotation with gene ontology terms in GOAT.. ACM SIGMOD Record.

